# Changes in Opioid-Involved Overdose Deaths by Opioid Type and Presence of Benzodiazepines, Cocaine, and Methamphetamine — 25 States, July–December 2017 to January–June 2018

**DOI:** 10.15585/mmwr.mm6834a2

**Published:** 2019-08-30

**Authors:** R. Matt Gladden, Julie O’Donnell, Christine L. Mattson, Puja Seth

**Affiliations:** 1 Division of Unintentional Injury Prevention, National Center for Injury Prevention and Control, CDC.

From 2013 to 2017, the number of opioid-involved overdose deaths (opioid deaths) in the United States increased 90%, from 25,052 to 47,600.* This increase was primarily driven by substantial increases in deaths involving illicitly manufactured fentanyl (IMF) or fentanyl analogs^†^ mixed with heroin, sold as heroin, or pressed into counterfeit prescription pills (*1*–*3*). Methamphetamine-involved and cocaine-involved deaths that co-involved opioids also substantially increased from 2016 to 2017 (*4*). Provisional 2018^§^ estimates of the number of opioid deaths suggest a small decrease from 2017. Investigating the extent to which decreases occurred broadly or were limited to a subset of opioid types (e.g., prescription opioids versus IMF) and drug combinations (e.g., IMF co-involving cocaine) can assist in targeting of intervention efforts. This report describes opioid deaths during January–June 2018 and changes from July–December 2017 in 25^¶^ of 32 states and the District of Columbia participating in CDC’s State Unintentional Drug Overdose Reporting System (SUDORS).** Opioid deaths were analyzed by involvement (opioid determined by medical examiner or coroner to contribute to overdose death) of prescription or illicit opioids,^††^ as well as by the presence (detection of the drug in decedent) of co-occurring nonopioid drugs (cocaine, methamphetamine, and benzodiazepines). Three key findings emerged regarding changes in opioid deaths from July–December 2017 to January–June 2018. First, overall opioid deaths decreased 4.6%. Second, decreases occurred in prescription opioid deaths without co-involved illicit opioids and deaths involving non-IMF illicit synthetic opioids (fentanyl analogs and U-series drugs) (*5*). Third, IMF deaths, especially those with multiple illicit opioids and common nonopioids, increased. Consequently, IMF was involved in approximately two-thirds of opioid deaths during January–June 2018. Notably, during January–June 2018, 62.6% of all opioid deaths co-occurred with at least one common nonopioid drug. To maintain and accelerate reductions in opioid deaths, efforts to prevent IMF-involved deaths and address polysubstance misuse with opioids must be enhanced. Key interventions include broadening outreach to groups at high risk for IMF or fentanyl analog exposure and overdose. Improving linkage to and engagement in risk-reduction services and evidence-based treatment for persons with opioid and other substance use disorders with attention to polysubstance use or misuse is also needed.

Numbers of opioid deaths of unintentional and undetermined intent^§§^ occurring during January–June 2018 and changes from July–December 2017 were analyzed for 25 of the 32 states and the District of Columbia that participate in SUDORS (data for these periods were the most recent and complete). The states abstract death certificate and medical examiner and coroner report data, including death scene investigation and toxicology findings. States list drugs involved in (i.e., contributing to) the opioid death as determined by medical examiners and coroners^¶¶^ and all drugs detected (present or co-occurring) by toxicologic tests. Fentanyl and morphine deaths were classified as prescription opioid deaths or illicit opioid deaths based on scene evidence and toxicology findings.*** Changes in the number of opioid deaths from July–December 2017 to January–June 2018 were analyzed by five opioid types^†††^: 1) prescription, 2) IMF, 3) fentanyl analog, 4) heroin, and 5) U-series. Because the frequency and changes in opioid deaths might vary by co-involvement with IMF or other illicit opioids, opioid deaths were also grouped into the following eight mutually exclusive categories: 1) IMF with no other illicit opioids involved; 2) IMF co-involving heroin; 3) IMF co-involving fentanyl analogs; 4) co-involved IMF, heroin, and fentanyl analogs; 5) heroin with no other illicit opioids involved; 6) fentanyl analogs with no other illicit opioids involved; 7) prescription opioids with no illicit opioids involved; and 8) all other opioid combinations. Finally, deaths were analyzed by nonopioids (cocaine, methamphetamine, and benzodiazepines) that are commonly present and involved in opioid deaths.^§§§^ Tracking the presence of commonly occurring nonopioids is important to inform public health action and has implications for treatment approaches. Some opioid deaths were grouped into one or more of the five opioid type categories and nonopioid drug combinations because multiple opioids and nonopioids might be involved in a single death (e.g., an opioid death involving IMF, heroin, cocaine, and a benzodiazepine). Changes in numbers of opioid deaths over the analysis period were tested using z-tests or nonoverlapping confidence intervals if the number of deaths was <100. SAS statistical software (version 9.4; SAS Institute, Inc.) was used for all analyses; p-values <0.05 were considered statistically significant.^¶¶¶^

During January–June 2018, among 13,631 opioid deaths in the 25 states, data on specific opioids involved were available for 13,415 (98.4%). IMF was co-involved in 68.0% of 5,281 heroin deaths and most (82.1%) of 2,678 fentanyl analog deaths (Table 1). In addition, 1,562 (40.5%) of 3,853 prescription opioid deaths co-involved illicit opioids. Opioids commonly involved in opioid deaths were IMF (67.9%), heroin (39.4%), prescription opioids (28.7%), and fentanyl analogs (20.0%) (Table 2). Among categories of deaths involving IMF, those with no other illicit opioids involved, those co-involved with heroin, those co-involved with fentanyl analogs, and those co-involved with heroin and fentanyl analogs accounted for 32.2%, 19.1%, 8.7%, and 7.5% of deaths, respectively. Heroin without other illicit opioids involved accounted for 11.4% of deaths, fentanyl analogs with no other illicit opioids involved for 2.3%, prescription opioids with no illicit opioids involved for 17.1%, and all other opioid combinations for 1.6%. In the Midwest, Northeast, and South U.S. Census regions, deaths involving any IMF were more common than were those involving any heroin. In the West, heroin-involved deaths (47.5%) were more common than were IMF-involved deaths (15.8%) (data not shown).

**TABLE 1:** Number and percentage of opioid overdose deaths that co-involved another opioid, by opioid type (illicitly manufactured fentanyl [IMF],* fentanyl analogs,^†^ heroin, and prescription opioids^§^) — 25 states,^¶^ State Unintentional Drug Overdose Reporting System (SUDORS), January–June 2018

Opioid type involved in opioid death**	No. of deaths Jan–Jun 2018	No. of deaths with co-involved opioid types (%)				
		IMF	Fentanyl analog	Heroin	Other illicit opioid^††^	Prescription opioid
Any suspected IMF	9,105	—	2,199 (24.2)	3,589 (39.4)	4,785 (52.6)	1,250 (13.7)
Any fentanyl analog	2,678	2,199 (82.1)	—	1,172 (43.8)	2,366 (88.3)	356 (13.3)
Any suspected heroin	5,281	3,589 (68.0)	1,172 (22.2)	—	3,747 (71.0)	796 (15.1)
Any prescription opioid	3,853	1,250 (32.4)	356 (9.2)	796 (20.7)	1,562 (40.5)	—

*Among fentanyl-involved deaths, 87.2%, 11.2%, and 1.6% were suspected to involve IMF, had insufficient data to classify the fentanyl death as IMF or prescription fentanyl, and were suspected to involve prescription fentanyl, respectively. Because the majority of identified cases involved IMF, and characteristics of unclassified fentanyl deaths were more similar to IMF-involved deaths than to prescription fentanyl–involved deaths, unclassified fentanyl deaths were categorized as suspected IMF-involved.

^†^ Fentanyl analog-involved deaths included deaths involving carfentanil, acetylfentanyl, acrylfentanyl, furanylfentanyl, 3-methylfentanyl, butyrylfentanyl, cyclopropylfentanyl, crotonylfentanyl, 4/para-fluorofentanyl, 4/para-fluorobutyrylfentanyl, 4/para-isobutyrylfentanyl, cyclopentylfentanyl, methoxyacetylfentanyl, isobutyrylfentanyl, furanylethylfentanyl, methoxybutyrlfentanyl, benzylfentanyl, valerylfentanyl, alpha-methylfentanyl, tetrahydrofuranylfentanyl, ocfentanil, betahydroxythiofentanyl, alfentanil, sufentanil, methylcarfentanil, methylthiofentanyl, phenylfentanyl, omethylacetylfentanyl, and isovalerylfentanyl.

^§^ Included any opioid deaths involving prescription opioids (oxycodone, oxymorphone, hydrocodone, hydromorphone, tramadol, buprenorphine, methadone, morphine, codeine, prescription fentanyl, meperidine, tapentadol, dextrorphan, levorphanol, propoxyphene, noscapine, and pentazocine). Other drugs might have been involved or co-occurred with the prescription opioid.

^¶^ Alaska, Connecticut, Delaware, Florida, Georgia, Illinois, Kentucky, Maine, Massachusetts, Minnesota, Missouri, Nevada, New Jersey, New Mexico, North Carolina, Ohio, Oklahoma, Pennsylvania, Rhode Island, Tennessee, Utah, Vermont, Virginia, Washington, and Wisconsin.

** U-series deaths were not reported in the analyses because only 63 deaths involved U-series drugs in the 25 SUDORS states during January–June 2018.

^††^ Any illicit opioid other than that listed in drug category. For deaths involving illicit opioids (IMF, fentanyl analogs, and heroin) the illicit drug is excluded from this column. For example, 52.6% of IMF deaths co-involved at least one fentanyl analog, heroin, or U-series drug.

**TABLE 2:** Change in the number of opioid overdose deaths, by opioid type, eight common opioid drug combinations, and commonly co-occurring nonopioids (cocaine, methamphetamine, and benzodiazepines) — 25 states,* State Unintentional Drug Overdose Reporting System (SUDORS), July–December 2017 to January–June 2018

Characteristic	Opioid deaths with information on involved opioids, Jan–Jun 2018, no. (%)	Change in no. of opioid deaths, Jul–Dec 2017 to Jan–Jun 2018, no. (%)	% Nonopioid drugs commonly present in opioid deaths, Jan–Jun 2018			
			Cocaine, any^†^	Meth-amphetamine, any^†^	Benzo-diazepines, any	All three drugs, any
**Total opioid overdose deaths**	**13,415 (100)**	**−648 (−4.6)^§^**	**34.0**	**12.1**	**32.5**	**62.6**
**% of deaths with contributing nonopioid drugs present^¶^**	NA	NA	81.3	81.8	67.5	80.6**
**Opioid drug class or drug involved in opioid deaths^††^**						
Any prescription opioid^§§^	3,853 (28.7)	−271 (−6.6)^§^	19.8	10.0	50.1	64.3
Any illicit opioid^¶¶^	11,124 (82.9)	−376 (−3.3)^§^	38.6	12.8	28.2	62.6
Any suspected IMF***	9,105 (67.9)	910 (11.1)^§^	39.7	11.2	27.8	62.0
Any suspected heroin	5,281 (39.4)	−83 (−1.5)	38.4	13.8	28.5	63.1
Any fentanyl analog^†††^	2,678 (20.0)	−627 (−19.0)^§^	40.3	11.2	30.9	63.6
Any U-series^§§§^	63 (0.5)	−190 (−75.1)^§^	36.5	7.9	39.7	61.9
**Common mutually exclusive combinations of opioids involved in opioid deaths^¶¶¶^**						
Opioid combinations co-involving IMF						
IMF with no other illicit opioids	4,320 (32.2)	370 (9.4)^§^	38.3	12.1	27.1	61.7
IMF with heroin	2,566 (19.1)	222 (9.5)^§^	40.8	9.2	26.9	61.0
IMF with fentanyl analogs	1,172 (8.7)	120 (11.4)^§^	41.6	11.8	29.6	64.2
IMF with heroin and fentanyl analogs	1,008 (7.5)	250 (33.0)^§^	40.4	11.8	30.5	63.4
Illicit opioid combinations not co-involving IMF						
Heroin with no other illicit opioid	1,534 (11.4)	−306 (−16.6)^§^	33.3	23.5	28.9	66.4
Fentanyl analogs with no other illicit opioid	312 (2.3)	−661 (−67.9)^§^	35.9	9.6	33.3	61.5
Prescription opioid with no illicit opioid	2,291 (17.1)	−272 (−10.6)^§^	11.6	8.7	53.5	62.6
All other combinations of opioids	212 (1.6)	−371 (−63.6)^§^	36.3	7.1	36.3	61.8
**Fentanyl analog deaths by acetylfentanyl and IMF co-involvement**						
Any acetylfentanyl	1,716 (12.8)	590 (52.4)^§^	40.9	12.0	29.5	63.6
Acetylfentanyl with IMF	1,685 (12.6)	615 (57.5)^§^	41.0	12.0	29.3	63.5
Acetylfentanyl no IMF	31 (0.2)	−25 (−44.6)	35.5	9.7	41.9	67.7
All other fentanyl analogs	1,100 (8.2)	−1,228 (−52.7)^§^	39.8	9.8	32.4	63.5
Other fentanyl analogs with IMF	645 (4.8)	−274 (−29.8)^§^	42.9	10.9	30.7	64.8
Other fentanyl analogs no IMF	455 (3.4)	−954 (−67.7)^§^	35.4	8.4	34.7	61.8

**Abbreviations**: IMF = illicitly manufactured fentanyl; NA = not applicable.

* Alaska, Connecticut, Delaware, Florida, Georgia, Illinois, Kentucky, Maine, Massachusetts, Minnesota, Missouri, Nevada, New Jersey, New Mexico, North Carolina, Ohio, Oklahoma, Pennsylvania, Rhode Island, Tennessee, Utah, Vermont, Virginia, Washington, and Wisconsin.

^†^ Only the two most frequently co-occurring types of stimulants (cocaine and methamphetamine) are reported because other types of stimulants such as amphetamines did not meet inclusion criteria.

^§^ Statistically significantly change from July–December 2017 to January–June 2018 based on z-tests or nonoverlapping confidence intervals if the number of deaths was <100 (p<0.05).

^¶^ For cocaine, methamphetamine, and benzodiazepines, this row reports a percentage calculated by dividing the number of opioid deaths in which the drug was present and reported as contributing to the opioid death (numerator) by the number of opioid deaths in which the drug was present (i.e., detected by toxicology tests) irrespective of whether it contributed to the opioid death (denominator).

** Percentage of all opioid deaths in which cocaine, methamphetamine, or benzodiazepines contributed to death.

^††^ An opioid death might involve multiple opioids. Thus, total opioid deaths and change in opioid deaths will be different than the sum of the deaths associated with each opioid type. Other nonopioid drugs might have been involved or co-occurred.

^§§^ Included any opioid death involving prescription opioids (oxycodone, oxymorphone, hydrocodone, hydromorphone, tramadol, buprenorphine, methadone, morphine, codeine, prescription fentanyl, meperidine, tapentadol, dextrorphan, levorphanol, propoxyphene, noscapine, and pentazocine). Other drugs might have been involved or co-occurred.

^¶¶^ Included any opioid death involving IMF, heroin, fentanyl analogs, or U-series drugs. Other drugs might have been involved or co-occurred.

*** Among fentanyl-involved deaths, 87.2%, 11.2%, and 1.6% were suspected to involve IMF, had insufficient data to classify the fentanyl death as IMF or prescription fentanyl, and were suspected to involve prescription fentanyl, respectively. Because the majority of identified cases involved IMF, and characteristics of unclassified fentanyl deaths were more similar to IMF-involved deaths than to prescription fentanyl–involved deaths, unclassified fentanyl deaths were categorized as suspected IMF-involved.^†††^ Fentanyl analog deaths included deaths involving carfentanil, acetylfentanyl, acrylfentanyl, furanylfentanyl, 3-methylfentanyl, butyrylfentanyl, cyclopropylfentanyl, crotonylfentanyl, 4/para-fluorofentanyl, 4/para-fluorobutyrylfentanyl, 4/para-isobutyrylfentanyl, cyclopentylfentanyl, methoxyacetylfentanyl, isobutyrylfentanyl, furanylethylfentanyl, methoxybutyrlfentanyl, benzylfentanyl, valerylfentanyl, alpha-methylfentanyl, tetrahydrofuranylfentanyl, ocfentanil, betahydroxythiofentanyl, alfentanil, sufentanil, methylcarfentanil, methylthiofentanyl, phenylfentanyl, omethylacetylfentanyl, and isovalerylfentanyl.

^§§§^ U-series drugs are novel nonfentanyl-related synthetic opioids with no authorized medical uses. U-series drug deaths include those involving U-47700 and its analogs U-48800 and U-49900. U-47700, a nonfentanyl benzamide compound developed by a pharmaceutical company, is not authorized for medical use in the United States and is currently distributed illicitly for its heroin-like effect. Deaths involving U-50488 and U-51754 were also included in this category, but each was involved in five or fewer deaths.

^¶¶¶^ Six categories are combinations of the illicit opioids involved in death (IMF, heroin, fentanyl analog, and U-series) that were involved in >200 deaths during January–June 2018. These deaths might co-involve prescription opioids and co-occur with nonopioids. The “prescription opioids with no illicit opioid” category includes only deaths involving prescription opioids with no illicit opioid co-involvement but might co-occur with other nonopioid drugs. The “all other combinations of opioids” category includes opioid deaths that involved opioid drug combinations not listed, primarily opioid deaths involving U-series drugs or heroin deaths co-involving fentanyl analogs.

Three principal changes occurred in opioid deaths from July–December 2017 to January–June 2018. First, overall opioid deaths in the 25 states declined by 4.6% (Table 2). Second, declines occurred in prescription opioid deaths with no co-involved illicit opioids (10.6%) and non-IMF illicit synthetic opioid deaths, including fentanyl analogs (19.0% decline) and U-series drugs (75.1% decline). With the exception of acetylfentanyl, decreases in fentanyl analog deaths occurred broadly across all fentanyl analogs (52.7% decline). Acetylfentanyl deaths co-involving IMF showed a sharp increase (57.5%). Third, IMF deaths increased by 11.1% overall, with increases of 9.5%–33.0% in those co-involving other illicit opioids and 9.4% among those with no other illicit opioids involved. Illicit opioid overdose deaths involving heroin and fentanyl analogs increased when IMF was co-involved, but decreased when IMF and other illicit opioids were not co-involved. Specifically, increases occurred in IMF deaths co-involving heroin (9.5%), fentanyl analogs (11.4%), and both heroin and fentanyl analogs (33.0%). In contrast, substantial declines were observed in heroin deaths with no other illicit opioids involved (16.6% decline) and fentanyl analog deaths with no other illicit opioids involved (67.9% decline). Declines in heroin deaths with no other illicit opioids involved were offset by increases in heroin deaths co-involving IMF, resulting in no significant change in heroin deaths.

The majority of opioid deaths (62.6%) co-occurred with one or more of the following drugs: benzodiazepines, cocaine, and methamphetamine, which were each present in 32.5%, 34.0%, and 12.1% of deaths, respectively. From July–December 2017 to January–June 2018, opioid deaths without benzodiazepines, cocaine, or methamphetamine decreased 8.0%, and opioid deaths co-occurring with benzodiazepines significantly decreased 5.7% (Table 3). Conversely, opioid deaths co-occurring with methamphetamine significantly increased by 14.6%. IMF deaths that co-occurred with benzodiazepines, cocaine, and methamphetamine significantly increased from July–December 2017 to January–June 2018 by 11.3%, 14.0%, and 31.0%, respectively, as IMF deaths without benzodiazepines, cocaine, or methamphetamine increased 6.7%.

**TABLE 3:** Changes in the number and percentage of opioid deaths co-occurring with benzodiazepines, cocaine, and methamphetamine, by type of opioids involved in death — 25 states,* State Unintentional Drug Overdose Reporting System (SUDORS), July–December 2017 to January–June 2018

Type of opioid involved in death	No. of opioid deaths with co-occurring drugs (%)			
	Benzodiazepines^†^	Cocaine^†^	Methamphetamine^†^	None of the three drugs
**All opioids^§^**	**−264 (−5.7)^¶^**	**−106 (−2.3)**	**206 (14.6)^¶^**	**−437 (−8.0)^¶^**
Any IMF**	256 (11.3)^¶^	445 (14.0)^¶^	241 (31.0)^¶^	217 (6.7)^¶^
Illicit opioid, no IMF^††^	−389 (−39.0)^¶^	−514 (−42.9)^¶^	−69 (−14.7)^¶^	−537 (−43.3)^¶^
Prescription opioid, no illicit opioid^§§^	−131 (−9.7)^¶^	−37 (−12.2)	34 (20.5)	−117 (−12.0)^¶^

**Abbreviation:** IMF = illicitly manufactured fentanyl.

* Alaska, Connecticut, Delaware, Florida, Georgia, Illinois, Kentucky, Maine, Massachusetts, Minnesota, Missouri, Nevada, New Jersey, New Mexico, North Carolina, Ohio, Oklahoma, Pennsylvania, Rhode Island, Tennessee, Utah, Vermont, Virginia, Washington, and Wisconsin.

^†^ Opioid deaths co-occurring with benzodiazepines, cocaine and methamphetamine are not mutually exclusive as deaths associated with multiple nonopioids (e.g., cocaine and benzodiazepines) will be counted in both categories.

^§^ All opioid deaths (N = 13,415 during January–June 2018).

^¶^ Statistically significant change from July–December 2017 to January–June 2018 based on z-tests or nonoverlapping confidence intervals if the number of deaths was <100 (p<0.05).

** All opioid deaths where IMF was involved (N = 9,105 during January–June 2018).

^††^ All deaths involving other illicit opioids (heroin, fentanyl analogs, and U-series) where IMF was not involved (N = 2,019 during January–June 2018).

^§§^ Deaths involving prescription opioids without illicit opioid involvement (N = 2,291 deaths during January–June 2018).

## Discussion

Among 25 states participating in SUDORS, three major changes in opioid deaths from July–December 2017 to January–June 2018 were identified. These included 1) overall decreases in opioid overdose deaths; 2) decreases in both prescription opioid deaths without co-involved illicit opioids and non-IMF illicit synthetic opioids (i.e., fentanyl analogs and U-series drugs) deaths; and 3) increases in IMF deaths, especially those with heroin, fentanyl analogs or nonopioid drugs. Also, at least one nonopioid drug (benzodiazepines, cocaine, or methamphetamine) was present in the majority of opioid deaths during January–June 2018. Prescription opioid deaths stabilized nationally from 2016 to 2017 (*6*), and the number of opioid prescriptions filled has been decreasing for several years,**** as efforts to reduce high-risk prescribing have increased. Findings from this report suggest these efforts might have fostered decreases in prescription opioid deaths without illicit opioids.

This report is one of the first to document large decreases in fentanyl analog and U-series drug deaths across multiple states, including decreases in deaths involving fentanyl analogs (e.g., carfentanil) that drove local outbreaks during 2016–2017.^††††^ In contrast, rapid increases in deaths co-involving acetylfentanyl and IMF might partly result from unintentional production of acetylfentanyl at very low levels during the IMF manufacturing process, rather than deliberate mixing or co-use of acetylfentanyl with IMF (*7*). Substantial decreases in fentanyl analog and U-series drug deaths even as IMF deaths continue to increase suggest supply changes requiring investigation.

Although concerning, the 6-month 11.1% increase in IMF deaths in the 25 states is smaller than the approximate doubling of U.S. fentanyl deaths each year during 2014–2016.^§§§§^ Because IMF is distributed primarily in the powder heroin market,^¶¶¶¶^ slower increases in IMF deaths might reflect successes in one or more objectives: reducing the number of persons who initiate heroin use, increasing treatment access for persons misusing heroin,***** expanding naloxone access,^†††††^ or changing behaviors of persons injecting drugs to reduce the likelihood of an IMF-involved overdose (*8*).

Evidence suggests that persons using powdered heroin are often unaware of whether IMF or fentanyl analogs are present in illicit products (*2*,*3*). Consequently, IMF, heroin, and fentanyl analog combinations in opioid deaths might represent mixed drug products rather than purposeful co-use. IMF deaths without other illicit opioids co-involved and IMF deaths co-involving heroin are the two most frequent drug combinations in opioid deaths and are consistent with combinations found when drug products test positive for fentanyl by law enforcement (*2*). As IMF supply expanded during January–June, 2018 (*9*), a large, nationally accredited laboratory reported that the majority of patients east of the Mississippi River who tested positive for heroin also tested positive for fentanyl, and this percentage increased in early 2018.^§§§§§^ This suggests increased mixing of IMF with powdered heroin and fewer heroin-only products, consistent with the increases in IMF deaths co-involving heroin and decreases in heroin deaths without IMF documented in this report. In Western states, heroin deaths predominated, possibly because of the limited mixing of powdered IMF in black tar heroin, which is distributed primarily in the West (*1*,*3*). Continued vigilance is needed because synthetic opioid (excluding methadone [likely fentanyl]) deaths increased in eight states west of the Mississippi in 2017 (*6*), and the IMF supply in the West increased during the first half of 2018 (*9*). Finally, increased distribution of counterfeit prescription pills that contain IMF might increase the risk of overdose in persons who use prescription medications not prescribed to them, especially opioid pain relievers (*2*).^¶¶¶¶¶^

The majority of opioid deaths co-occurred with benzodiazepines, cocaine, or methamphetamine highlighting the need to address polysubstance use in the prevention of overdoses and treatment of opioid misuse. Increases in opioid deaths, especially IMF deaths, co-occurring with methamphetamine are consistent with previous reports (*4*) and with increases in methamphetamine supply (*9*) and methamphetamine use among persons seeking treatment for opioid misuse (*10*). Moreover, IMF deaths co-occurring with benzodiazepines and cocaine increased during January–June 2018 even as overall opioid deaths co-occurring with benzodiazepines and cocaine decreased or did not significantly change, respectively. Increases in IMF deaths co-occurring with cocaine are consistent with previous reports (*4*) and with high co-use of cocaine among persons injecting heroin****** and outbreaks linked to rare but increasing numbers of drug products that mix IMF and cocaine (*2*).^††††††^

The findings in this report are subject to at least five limitations. First, toxicology testing and classification protocols vary over time and across jurisdictions, which affects whether drugs were detected and classified as contributing to death. Second, misclassification of prescription and illicit substances might occur, but this was minimized by using detailed toxicology results and scene evidence. Third, focus on drugs commonly involved in opioid deaths might obscure emerging drug issues. Fourth, patterns in drugs involved in opioid deaths might vary across states and demographic groups. Finally, findings are limited to the 25 states participating in SUDORS and might not be generalizable to other states.

Increases in IMF deaths involving multiple illicit opioids and benzodiazepines, cocaine, and methamphetamine (nonopioids) highlight the need to better understand how the risk of IMF overdose varies by illicit product potency, variation in potency, and form (e.g., powder or counterfeit pill) and a person’s tolerance or polysubstance use patterns. In response, CDC’s Overdose Data to Action funding^§§§§§§^ expands SUDORS from including only opioid-involved deaths to including all drug overdose deaths to better understand increases in IMF and stimulant and drug combination deaths (with and without opioids), as well as identify emerging threats. Key interventions include broadening outreach to groups at high risk for IMF or fentanyl analog exposure and overdose. Improving linkage to and engagement in risk-reduction services and evidence-based treatment for persons with opioid and other substance use disorders with attention to polysubstance use or misuse is also needed.

**Summary**

**What is already known about this topic?**

Provisional opioid-involved overdose deaths suggest slight declines from 2017 to 2018, contrasting with sharp increases during 2014–2017 driven by fentanyl overdose deaths.

**What is added by this report?**

From July–December 2017 to January–June 2018 in 25 states, opioid deaths decreased 5% overall and decreased for prescription opioids and illicit synthetic opioids excluding illicitly manufactured fentanyl (IMF). However, IMF deaths increased 11%. Benzodiazepines, cocaine, or methamphetamine were present in 63% of opioid deaths.

**What are the implications for public health practice?**

Continued increases in IMF deaths highlight the need to broaden outreach to persons at high risk for IMF overdoses and improve linkage to risk-reduction services and evidence-based treatment. Prevention and treatment efforts should attend to broad polysubstance use/misuse.

## Footnotes

* https://www.cdc.gov/nchs/data/databriefs/db329_tables-508.pdf#4.

† Fentanyl is a synthetic opioid 50–100 times more potent than morphine and is approved for treatment of severe (typically advanced cancer) pain. Illicitly manufactured fentanyl is manufactured illegally and sold through illegal drug markets for its heroin-like effect. Fentanyl analogs, also known as fentanyl-related substances, are synthetic opioids that are similar in chemical structure to fentanyl but modified to generate distinct substances. Fentanyl analogs vary in potency, with some more potent than fentanyl and others with potency similar to or less than fentanyl. https://www.cdc.gov/drugoverdose/opioids/fentanyl.html; https://www.deadiversion.usdoj.gov/drug_chem_info/frs.pdf.

§ https://www.cdc.gov/nchs/nvss/vsrr/drug-overdose-data.htm.

¶ Alaska, Connecticut, Delaware, Florida, Georgia, Illinois, Kentucky, Maine, Massachusetts, Minnesota, Missouri, Nevada, New Jersey, New Mexico, North Carolina, Ohio, Oklahoma, Pennsylvania, Rhode Island, Tennessee, Utah, Vermont, Virginia, Washington, and Wisconsin. Florida, Illinois, Missouri, Pennsylvania, and Washington collect data from a subset of counties that accounted for 77%–87% of all unintentional or undetermined-intent opioid overdoses in 2017.

** SUDORS captures detailed information on toxicology, death scene investigations, route of administration, and other risk factors that may be associated with a fatal overdose. SUDORS is part of CDC’s Enhanced State Opioid Overdose Surveillance (ESOOS) program (which funded 12 states through a competitive application process in Fiscal Year 2016 and an additional 20 states and the District of Columbia in Fiscal Year 2017). https://www.cdc.gov/drugoverdose/foa/state-opioid-mm.html.

†† IMF, fentanyl analogs, heroin, and illicitly manufactured U-series drugs. U-series drugs are novel nonfentanyl-related synthetic opioids with no authorized medical uses. U-series drug deaths include those involving U-47700 and its analogs U-48800 and U-49900. U-47700, a nonfentanyl benzamide compound developed by a pharmaceutical company, is not authorized for medical use in the United States and is currently distributed illicitly for its heroin-like effect. Deaths involving U-50488 and U-51754 were also included in this category, but each was involved in five or fewer deaths.

§§ A death was included in the analysis if 1) the fatal injury occurred within, and was reported by, one of the 25 SUDORS states, and 2) the death was classified as an overdose death involving opioids either through review of the medical examiner/coroner report or the death certificate had *International Classification of Diseases, Tenth Revision* underlying cause-of-death codes X40–44 (unintentional) or Y10–Y14 (undetermined intent) and multiple cause-of-death codes T40.0, T40.1, T40.2, T40.3, T40.4, or T40.6. Data for this report were downloaded on June 26, 2019, and might differ from earlier or future reports because states continually update death data and investigations of suspected drug overdose deaths might involve lengthy investigations.

¶¶ All substances detected were categorized as contributing to the death when deaths were classified by the medical examiner/coroner as a drug overdose and toxicology results were available, but no information was available on the specific drugs contributing to death.

*** Fentanyl was classified on the basis of toxicology, scene, and witness evidence that indicated the likely source as either prescription (e.g., scene evidence of fentanyl patches at the overdose location) or illicit (e.g., toxicology evidence of a fentanyl analog, or scene or witness evidence of illicit drug use including injection or snorting). If evidence of prescription or illicit use was not available, fentanyl was categorized as IMF because the vast majority of fentanyl overdose deaths involve IMF. Morphine in the absence of 6-acetylmorphine (a metabolite of heroin) was classified as likely prescription morphine (scene or witness evidence of prescription morphine use) or as likely heroin (toxicology evidence of heroin impurities or other illicit drug detected or scene or witness evidence that indicated injection, illicit drug use, or a history of heroin use). If evidence of prescription or illicit use was not available, morphine was categorized as prescription because the investigation did not obtain scene evidence of heroin use or detect 6-acetylmorphine. If morphine was detected along with 6-acetylmorphine, it was classified as heroin.

††† Substances coded as prescription opioids were oxycodone, oxymorphone, hydrocodone, hydromorphone, tramadol, buprenorphine, methadone, prescription fentanyl, morphine, codeine, meperidine, tapentadol, dextrorphan, levorphanol, propoxyphene, noscapine, and pentazocine. Also included as prescription drugs were brand names (e.g., Opana) and metabolites (e.g., nortramadol) of these substances and combinations of these substances and nonopioids (e.g., acetaminophen-oxycodone). Substances coded as illicit opioids were IMF, heroin, fentanyl analogs, and other illicit synthetic opioids, such as U-series drugs (e.g., U-47700), AH-7921, and MT-45. This analysis does not distinguish between prescription drugs prescribed to the decedent and those that were diverted. Data on AH-7921 and MT-45 were not included because they were involved in fewer than five deaths.

§§§ Only commonly occurring (present in >10% of opioid deaths) and contributing (involved in >50% of opioid deaths in which present) nonopioids were included. Cutoff for nonopioid inclusion was determined by a review of the data.

¶¶¶ To verify that changes in population size did not account for observed changes, sensitivity analyses were conducted in which crude population death rates per 100,000 residents for July–December 2017 were compared with death rates for January–June 2018 using z-tests or nonoverlapping confidence intervals if the number of deaths was <100. U.S. Department of Commerce’s Bureau of Economic Analysis data were used to estimate populations for the 25 states in the middle of the last quarter of 2017 and the middle of the second quarter of 2018 (https://apps.bea.gov/iTable/index.cfm/). For states collecting data on opioid deaths in a subset of counties, the estimates of state population were used and should have introduced minimal bias because of the >75% coverage of opioid deaths in these states and minimal population changes over a 6-month period.

****
https://www.cdc.gov/drugoverdose/pdf/pubs/2018-cdc-drug-surveillance-report.pdf.

†††† https://emergency.cdc.gov/han/han00413.asp.

§§§§ https://www.cdc.gov/nchs/data/nvsr/nvsr67/nvsr67_09-508.pdf.

¶¶¶¶ https://www.dea.gov/sites/default/files/2018-07/PRB-DIB-003-18.pdf.

***** https://www.hhs.gov/about/news/2018/09/14/samhsa-annual-mental-health-substance-use-data-provide-roadmap-for-future-action.html.

††††† https://www.cdc.gov/vitalsigns/naloxone/pdf/vs-0806-naloxone-H.pdf.

§§§§§ https://resource.millenniumhealth.com/signalsreportPR?from=signalsreportPR.

¶¶¶¶¶ https://www.dea.gov/sites/default/files/2018-07/PRB-DIB-003-18.pdf.

****** https://www.cdc.gov/hiv/pdf/library/reports/surveillance/cdc-hiv-hssr-nhbs-pwid-2015.pdf.

†††††† https://emergency.cdc.gov/han/han00413.asp.

§§§§§§ https://www.cdc.gov/drugoverdose/od2a/index.html.
